# The synergistic effects of applying low-level laser therapy plus ultrasound on pain and muscle function in patients with knee osteoarthritis: A protocol of a randomized double-blind study

**DOI:** 10.1097/MD.0000000000024764

**Published:** 2021-03-12

**Authors:** Sihong Li, Min Yang, Long Tang, Yizhao Zhou

**Affiliations:** a Department of orthopedics, Hunan Provincial People's Hospital; b Department of Gerontology, Changsha Central Hospital; c Department of orthopedics, Traditional Chinese Medicine of Huarong County, Hunan, China.

**Keywords:** double-blind, knee osteoarthritis, low-level laser therapy, protocol, randomized, ultrasound

## Abstract

**Background:**

To our knowledge, only 1 study with limited sample size tried to evaluate the synergistic effects of ultrasound and low-level laser therapy (LLLT) in patients with knee osteoarthritis. Further research is needed to confirm this synergy with larger numbers and better design. Therefore, we will conduct this present randomized double-blind study to evaluate the synergistic effects of simultaneously applying ultrasound plus LLLT on pain and muscle function in patients with knee osteoarthritis.

**Methods:**

The study protocol is a randomized, controlled, double-blind design. The study will be conducted at our academic hospital from February 2021 to January 2022. The study protocol was approved through Institutional Review Board in the Hunan Provincial People's Hospital. Patients will be assigned at random to the ultrasound + LLLT group, LLLT group, or the ultrasound group. After baseline examination, all patients will be given a full explanation of the treatment protocol and will be required to sign a written informed consent for study participation and for publication of the results. All the data collectors, surgeons, statistical analysts, as well as result assessors are not aware of grouping assignment. The primary outcome is weekly change in pain intensity relative to baseline through 6 weeks of therapy.

**Results:**

This protocol will provide a reliable theoretical basis for the following research.

**Conclusion:**

It is assumed that there will be a remarkable difference in postoperative outcomes between the intervention and control groups.

**Trial registration:**

This study protocol was registered in Research Registry (researchregistry6470).

## 1 Introduction

Osteoarthritis of the knee is a degenerative inflammatory disease that affects the entire joint and is characterized by progressive loss of cartilage with pain, disability, and decreased quality of life. The disease occurs when the dynamic equilibrium between deterioration and restoration becomes unbalanced, often in situations where the mechanical stress applied is greater than the one that can be supported by the joint tissues.^[1]^ Increased inflammatory activity is associated with higher pain intensity and faster disease progression in osteoarthritis.^[2]^

There are still no disease-modifying treatments for knee osteoarthritis. Currently, available options include palliative pharmacological and nonpharmacological modalities. The core goals of these therapies are to reduce joint pain, improve joint function, and achieve a better quality of life.^[3,4]^ Though nonsteroidal anti-inflammatory drugs are widely used to treat these patients, their side effects, particularly the high incidence of side effects in the upper gastrointestinal tract, limit their use. As a result, many physical therapy agents have been introduced, such as ultrasound, electrical stimulation, intensive exercise, and thermal therapy.^[5]^

Physical therapy is designed to reduce pain, improve function or quality of life, reduce the burden on the joint, promote adaptability to certain activities, prevent deformities and slow the progression of the disease. Jamtvedt et al performed a systematic review related to physical therapy for knee osteoarthritis and found that only exercise and weight loss were effective in improving pain and function.^[6]^ Acupuncture, percutaneous electrical nerve stimulation, and low-level laser therapy (LLLT) had moderate quality of evidence for the same variables. The quality of evidence for other interventions was low or nonexistent.^[7]^

Various studies have shown that LLLT has a positive therapeutic effect in treatment of knee osteoarthritis for rats, rabbits and clinical trials of humans.^[8–10]^ In parallel, therapeutic ultrasound has also shown positive effects for the treatment of knee osteoarthritis, both in vitro models and in vivo studies on animals and humans.^[11,12]^ The main positive effects of these modalities are, for example, anabolic effects on cartilage, anti-inflammatory effects, analgesia associated with muscle relaxation and improvements in microcirculation.

To our knowledge, only Paolillo et al tried to evaluate the synergistic effects of ultrasound and LLLT in patients with knee osteoarthritis. However, the sample size of their study was too small to draw convincing conclusions, thus further research is needed to confirm the synergies.^[13]^ Therefore, we will conduct this present randomized double-blind study to evaluate the synergistic effects of simultaneously applying ultrasound plus LLLT on pain and muscle function in patients with knee osteoarthritis. It is assumed that there will be a remarkable difference in postoperative outcomes between the intervention and control groups.

## 2 Materials and methods

### 2.1 Participants

Subjects will be included in this study if they

1. have painful knee osteoarthritis for at least 6 months with degenerative osteoarthritic knee of grade 2 to 3 or less based on radiographic diagnosis in the Kellgren and Lawrence grading of osteoarthritis,
2. have no limitation of range of motion (ROM) except for minimum tightness in the knee joint,
3. do not engage in any high-joint-loading exercises such as hiking or tennis playing and have not undergone any specific treatments 3 months before entering the study,
4. have a minimum score of 25 on the Western Ontario and McMaster Universities Osteoarthritis Index (WOMAC) total score, and
5. have a knee pain ≥4 on the visual analog scale (VAS) in the previous 3 months.

Subjects will be excluded from the study at pretreatment evaluation if they have any other musculoskeletal problems associated with the knee joint, such as fracture, tendon or ligament tears, meniscus injury, rheumatoid arthritis, or knee surgery. Patients will be also excluded if they have musculoskeletal problems associated with the hip or ankle/foot joints, have central or peripheral neuropathy, or have received physical therapy and/or intra-articular corticosteroid or hyaluronic acid injections during the last 6 months.

### 2.2 Study design

The study protocol is a randomized, controlled, double-blind design. The study will be conducted at our academic hospital from February 2021 to January 2022. The study protocol was approved through Institutional Review Board in the Hunan Provincial People's Hospital (HN20201208) and was registered in the research registry (with number: researchregistry6470).

### 2.3 Random allocation

**Figure 1:**
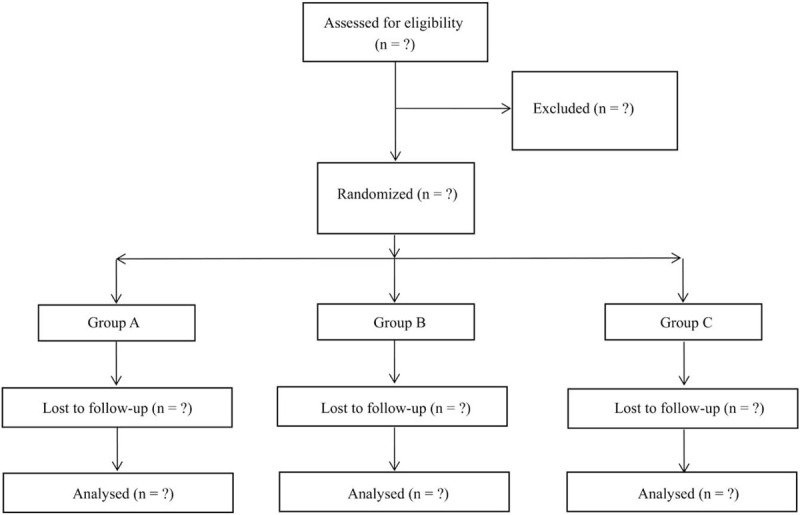
Consolidated Standards of Reporting Trials (CONSORT) diagram of patient flow through the study.

Before starting the study, a randomization list is produced using software-generated randomized numbers; the randomization depends on random blocks of 10 (Table 1). Patients will not know to which group they are assigned or which treatment they will be offered. Patients will be assigned at random to the ultrasound + LLLT group, LLLT group, or the ultrasound group. Participants are enrolled by the research assistant. After baseline examination, all patients will be given a full explanation of the treatment protocol and will be required to sign a written informed consent for study participation and for publication of the results. All the data collectors, surgeons, statistical analysts, as well as result assessors are not aware of grouping assignment (Fig. 1).

**Table 1:** Patient baseline demographics.

Demographics	Combined group	LLLT group	Ultrasound group	*P* value
Number of patients (knees)				
Age at surgery^∗^ (years)				
Male sex (no. [%])				
BMI^∗^ (kg/m^2^)				
Right side (no. [%])				
Follow-up^∗^ (years)				

BMI = body mass index, LLLT = low-level laser therapy.

∗ The values are given as the mean and the SD.

### 2.4 Intervention protocol

The interventions will be carried out twice a week for 2 months, for a total of 15 sessions, and will be performed by 2 experienced physiotherapists who are not evaluators, maintaining the blind nature of the method. For LLLT, the volunteers will be treated with low-level laser (Endolaser 476, Enraf Nonius, Rotterdam, The Netherlands), wavelength of 808 nm, 0.028 cm^2^ spot area, 100-mW power output, fluence of 200 J/cm^2^, energy per point of 5.6 J, in the regions of the lateral and medial epicondyle of tibia and femur, in the joint line of the lateral and medial knee, and the popliteal fossa (tendon of the biceps femoris, semitendinosus and between the tendons) and on the patellar tendon region, totaling 10 isolated points, for 56 seconds per point, total energy of 56 J. US will be applied using an aqueous gel as a coupling medium in circular movements with the probe at right angles. The treatment area is 25 cm^2^ and extends to both patellofemoral and tibiofemoral borders of the target knee on both the lateral and medial margins, avoiding the patella. Continuous ultrasonic waves with 1 MHZ frequency and 1 watt/cm^2^ power are applied with a 4-cm diameter applicator (Petson.250 ultrasound equipment Petas, Turkey) for 5 minutes in each session. To avoid the immediate effects of heat application, the outcome data evaluation is performed 2 days after completion of the last session.

### 2.5 Primary outcome

The primary outcome is weekly change in pain intensity relative to baseline through 6 weeks of therapy, as measured on the VAS (0 = no pain, 10 = extreme pain). The VAS pain scale has been validated for consistency and reliability to assess pain associated with a variety of conditions, including osteoarthritis. Patients records their pain 4 hours after applying the device during daily treatment.

### 2.6 Secondary outcomes

Secondary outcome measures assessed are change in WOMAC score for pain stiffness and function, ROM, and muscle strength assessed at baseline and the end of the study. ROM and strength measurements are acquired by trained clinic staff on a small pilot cohort of sequentially enrolled patients using computerized dual inclinometry ROM and muscle tester equipment (JTECH Medical, Midvale, UT). To assess ROM, 1 inclinometer is positioned on the quadricep muscle at mid femur and the other at mid-tibia. Patients are asked to lay flat on their back and lift their leg and flex at the knee to measure flexion. Patients are then asked to sit at the edge of the table and extend their leg to measure extension. To assess muscle strength, a manual muscle tester is used. Patients are asked to sit at the edge of the table with their knee at approximately 90 degrees. The manual muscle tester is positioned and hold at anterior mid tibia while patients are asked to extend their leg to measure extension strength. To measure flexion strength, the muscle tester is held at anterior mid-tibia and the patient is asked to flex at the knee. Each ROM and muscle strength test is repeated 3 times and the average is recorded (Table 2).

**Table 2:** Postoperative outcomes.

Outcomes	Combined group	LLLT group	Ultrasound group	*P* value
VAS				
WOMAC				
ROM				
Muscle strength				

LLLT = low-level laser therapy, ROM = range of motion, VAS = visual analog scale, WOMAC = Western Ontario and McMaster Universities Osteoarthritis Index.

### 2.7 Statistical analysis and power analysis

All analyses will be performed using SPSS for Windows, version 16, and GraphPad InStat. Analysis of variance is used for comparing mean values of patient's age, weight, height, and body mass index. For non-parametric measures like VAS and WOMAC, differences between baseline and post-treatment scores for each group are computed by the Wilcoxon signed ranks test. The difference between each treatment group is performed by the Kruskal–Wallis test. The level of statistical significance is set as *P* < .05. A power analysis is conducted to estimate the requisite sample size. From previous studies, a clinically significant difference reduction in the VAS was defined as 3 cm; a standard deviation of 3.49, probability of a Type I error of 0.05 and power of 0.8 resulted in an estimated sample size of 15. Consequently, a total of 120 subjects will be recruited.

## 3 Discussion

The most frequent treatment for knee osteoarthritis is prescription painkillers and anti-inflammatory medications. Serious health risks are associated with these medications, including addiction and increased risk for gastrointestinal, renal, and cardiovascular problems. Ultrasound has been used as a noninvasive and safe physiotherapy for musculoskeletal conditions since its initial approval by the Food and Drug Administration for the treatment of fractures in 1994.^[14]^ Unlike other treatments, therapeutic ultrasound not only relieves osteoarthritis patient symptoms but has potential cartilage healing effects. LLLT is another widely used electrophysical agent for the control of musculoskeletal pain and modulation of the inflammatory process.^[15]^ It reduces the IL1-β and COX-2, thus reducing prostaglandin E2 levels, besides biomodulation of osteomyoarticular changes in osteoarthritic process, through photochemical and photobiological properties.^[16]^

To our knowledge, only Paolillo et al tried to evaluate the synergistic effects of ultrasound and LLLT in patients with knee osteoarthritis. However, the sample size of their study was too small to draw convincing conclusions, thus further research is needed to confirm the synergies.^[13]^ Therefore, we will conduct this present randomized double-blind study to evaluate the synergistic effects of simultaneously applying ultrasound plus LLLT on pain and muscle function in patients with knee osteoarthritis. It is assumed that there will be a remarkable difference in postoperative outcomes between the intervention and control groups. In our protocol, a total of 120 subjects will be recruited.

## Author contributions

**Conceptualization:** Long Tang.

**Data curation:** Sihong Li.

**Formal analysis:** Sihong Li, Min Yang.

**Funding acquisition:** Yizhao Zhou.

**Investigation:** Sihong Li, Min Yang.

**Methodology:** Min Yang, Long Tang.

**Resources:** Yizhao Zhou.

**Software:** Sihong Li.

**Supervision:** Long Tang, Yizhao Zhou.

**Validation:** Min Yang.

**Visualization:** Long Tang.

**Writing – original draft:** Sihong Li.

**Writing – review & editing:** Yizhao Zhou.
